# Antibody sequence determinants of viral antigen specificity

**DOI:** 10.1128/mbio.01560-24

**Published:** 2024-09-12

**Authors:** Alexandra A. Abu-Shmais, Matthew J. Vukovich, Perry T. Wasdin, Yukthi P. Suresh, Toma M. Marinov, Scott A. Rush, Rebecca A. Gillespie, Rajeshwer S. Sankhala, Misook Choe, M. Gordon Joyce, Masaru Kanekiyo, Jason S. McLellan, Ivelin S. Georgiev

**Affiliations:** 1 Vanderbilt Vaccine Center, Vanderbilt University Medical Center, Nashville, Tennessee, USA; 2 Department of Pathology, Microbiology and Immunology, Vanderbilt University Medical Center, Nashville, Tennessee, USA; 3 Program in Chemical and Physical Biology, Vanderbilt University Medical Center, Nashville, Tennessee, USA; 4 Department of Computer Science, Vanderbilt University, Nashville, Tennessee, USA; 5 Department of Molecular Biosciences, The University of Texas at Austin, Austin, Texas, USA; 6 Vaccine Research Center, National Institute of Allergy and Infectious Diseases, National Institutes of Health, Bethesda, Maryland, USA; 7 Emerging Infectious Disease Branch, Walter Reed Army Institute of Research, Silver Spring, Maryland, USA; 8 Henry M. Jackson Foundation for the Advancement of Military Medicine, Inc., Bethesda, Maryland, USA; 9 Vanderbilt Institute for Infection, Immunology and Inflammation, Vanderbilt University Medical Center, Nashville, Tennessee, USA; 10 Center for Structural Biology, Vanderbilt University, Nashville, Tennessee, USA; 11 Program in Computational Microbiology and Immunology, Vanderbilt University Medical Center, Nashville, Tennessee, USA; McMaster University, Hamilton, Ontario, Canada

**Keywords:** B-cell responses, public clonotype, antigen specificity, virus, antibody repertoire, B-cell repertoire

## Abstract

Throughout life, humans experience repeated exposure to viral antigens through infection and vaccination, resulting in the generation of diverse, antigen-specific antibody repertoires. A paramount feature of antibodies that enables their critical contributions in counteracting recurrent and novel pathogens, and consequently fostering their utility as valuable targets for therapeutic and vaccine development, is the exquisite specificity displayed against their target antigens. Yet, there is still limited understanding of the determinants of antibody-antigen specificity, particularly as a function of antibody sequence. In recent years, experimental characterization of antibody repertoires has led to novel insights into fundamental properties of antibody sequences but has been largely decoupled from at-scale antigen specificity analysis. Here, using the LIBRA-seq technology, we generated a large data set mapping antibody sequence to antigen specificity for thousands of B cells, by screening the repertoires of a set of healthy individuals against 20 viral antigens representing diverse pathogens of biomedical significance. Analysis uncovered virus-specific patterns in variable gene usage, gene pairing, somatic hypermutation, as well as the presence of convergent antiviral signatures across multiple individuals, including the presence of public antibody clonotypes. Notably, our results showed that, for B-cell receptors originating from different individuals but leveraging an identical combination of heavy and light chain variable genes, there is a specific CDRH3 identity threshold above which B cells appear to exclusively share the same antigen specificity. This finding provides a quantifiable measure of the relationship between antibody sequence and antigen specificity and further defines experimentally grounded criteria for defining public antibody clonality.

**IMPORTANCE:**

The B-cell compartment of the humoral immune system plays a critical role in the generation of antibodies upon new and repeated pathogen exposure. This study provides an unprecedented level of detail on the molecular characteristics of antibody repertoires that are specific to each of the different target pathogens studied here and provides empirical evidence in support of a 70% CDRH3 amino acid identity threshold in pairs of B cells encoded by identical IGHV:IGL(K)V genes, as a means of defining public clonality and therefore predicting B-cell antigen specificity in different individuals. This is of exceptional importance when leveraging public clonality as a method to annotate B-cell receptor data otherwise lacking antigen specificity information. Understanding the fundamental rules of antibody-antigen interactions can lead to transformative new approaches for the development of antibody therapeutics and vaccines against current and emerging viruses.

## INTRODUCTION

The B-cell compartment of the adaptive immune system mediates a critical role in the generation of antibodies against invading pathogens (1). A principal feature of antibodies and their membrane-bound counterpart, the B-cell receptor (BCR), is the exquisite specificity displayed against cognate antigens. Indeed, the human antibody repertoire is subject to exceptional levels of diversity (2, 3), acting as an indispensable protective mechanism against the myriad of infectious diseases we may encounter throughout life. Yet still, the phenomenon of public antibody clonotypes—highly similar antibodies identified in multiple individuals—has been described in several settings (4–7), challenging this dogma of virtually unlimited antibody diversity.

Importantly, antigen specificity and therefore diversity of the antibody repertoire is the product of several complex and spatiotemporal mechanisms (3). Theoretical estimates of combinatorial and junctional diversity subjected to the naïve repertoire are upwards of 10^12^ BCR sequences and increase in orders of magnitude when considering the memory B-cell compartment (2, 8, 9). Upon antigen encounter, B cells travel to distal germinal centers and undergo cyclic rounds of somatic hypermutation resulting in the generation of highly specific antibodies that are able to recognize new and repeated pathogenic attacks. Generally, this is an idiosyncratic process; however, in the setting of a public antibody clonotype, this mechanism is occurring in a highly convergent manner.

BCR specificity can be defined by several sequence metrics, owing to the complex mechanisms described. Namely: germline variable (V), diversity (D), and joining (J) gene use, the length and amino acid composition of the complementarity determining region 3 (CDR3), the degree of somatic hypermutation across the heavy and light chain, the constant region isotype, and clonality. The precise definition of a public antibody clonotype can vary, with different restrictions on sequence identity and/or heavy and light chain gene usage proposed. A prevalent definition of “public” requires that clonotypes be present in more than one individual, sharing the same variable heavy (VH) genes and displaying highly similar CDRH3 regions, although with the advance of paired heavy-light chain sequencing, analogous restrictions on the variable light (VL) genes and CDRL3 sequences can also be added. Convergent antibody signatures have been reported in several anti-pathogen repertoires, including SARS-CoV-2 (10–13), human immunodeficiency virus 1 (HIV-1) (14), dengue (15, 16), respiratory syncytial virus (RSV) (17), ebola (18, 19), influenza (20–23), and others. The selective pressure toward expansion of a public clonotype suggests certain germline gene segments may have inherent features that facilitate recognition of viral proteins. Along this line, convergent sequence features identified within a specific antiviral repertoire, irrespective of public clonality, can lend further support to the notion of canonical molecular and structural features mediating recognition of viral antigenic targets.

Advances in repertoire sequencing (24–28) have enabled a greater understanding of the relationship between BCR sequence and antigen specificity, in the context of viral infectious disease. Particularly, since the onset of the COVID-19 pandemic, several studies have shed light on SARS-CoV-2-specific BCR signatures (10–13, 29–31). However, the vast majority of published BCR sequences in the public domain lack information about their cognate antigens, as even high-throughput antibody sequence identification methods, such as next-generation sequencing, are generally decoupled from the process of antibody functional characterization. As a result, while numerous insights into the innate properties of BCR sequences have been made, such as VH bias toward genes with increased genetic complexity (32, 33), light chain promiscuity as a result of decreased lambda/kappa theoretical diversity (34–36), and the concept of light chain coherence among public clonotypes (37), the impact of these phenomena in the presence of specific antigen activation and moreover, the similarities and differences of antibody responses to different viral pathogens, remains unclear.

In this study, leveraging a high-throughput B-cell discovery technology termed LIBRA-seq, linking B-cell receptor to antigen specificity, we systematically analyzed antigen-specific B-cell sequences against 20 viral antigens, representing several pathogens of current biomedical interest. Patterns in variable gene usage, gene pairing, CDRH3 length, and somatic hypermutation frequency were observed with respect to viral antigen specificity. Such patterns are evident when restricting analysis to B-cell breadth, as opposed to specificity, suggesting intrinsic differences exist between BCRs that display higher antigen binding selectivity, as opposed to those that are more widely cross-reactive against different antigens. Importantly, our results indicate that, for B-cell receptors originating from different individuals but expressing an identical combination of heavy and light chain variable genes, there is a specific CDRH3 identity threshold that defines whether these B cells may share the same antigen specificity, thereby providing new insights into the fundamental relationship of antibody sequence to antigen specificity, as well as a quantifiable threshold for defining antigen-specific public clonality.

## RESULTS

### Generation of antigen-specific B-cell receptor data sets by single-cell sequencing, bioinformatics, and recombinant validation of BCRs expressed as monoclonal antibodies

LIBRA-seq is a high-throughput antibody discovery tool that enables the simultaneous detection of B-cell receptor sequence and antigen specificity from a single biological sample. Here, the antigen screening library included 20 viral antigens, selected for their association with common infections and vaccinations, as well as a small subset of other antigens of current biomedical significance (Table 1). The library incorporated major antigenic targets for the selected pathogens, and in some cases, multiple strains per pathogen were included. Antigens have been grouped by viral family: Coronaviridae, Paramyxoviridae, Pneumoviridae, Orthomyxoviridae, and Flaviviridae. Sorted B cells displaying binding to HIV-1 BG505 Env, a negative control antigen that is not expected to be recognized in healthy individuals, were removed from all data sets on the basis of cell promiscuity.

**TABLE 1:** LIBRA-seq viral antigen screening library^*a*^

Viral family	Virus	Viral antigen	
Coronaviridae	Coronavirus	HKU1 spike	
		OC43 spike	
		SARS-CoV-2 spike	
Pneumoviridae	Respiratory syncytial virus (RSV)	RSV A fusion	
	Human metapneumovirus (hMPV)	RSV B fusion	
		hMPV A fusion	
		hMPV B fusion	
Paramyxoviridae	Human parainfluenza virus type 3 (hPIV3)	PIV 3 fusion	
Orthomyxoviridae	Influenza A	H1 MI/15 HA	H5 VN/04 HA
		H1 NC/99 HA	H7 AN/13 HA
		H3 Perth/19 HA	H9 HK/09 HA
		H3 HK/68 HA	H10 JD/13 HA
Flaviviridae	Dengue	DENV 1 E	
		DENV 2 E	
		DENV 3 E	
		DENV 4 E	
Retroviridae	Human immunodeficiency virus 1 (HIV-1)	HIV-1 BG505 env	

^*a*^ The screening library included antigens associated with common infections and vaccinations, as well as a selection of other antigens of biomedical significance. A total of 20 antigens representing multiple strains across five distinct viral families was included. This antigen library incorporates some of the major antigenic targets for the selected pathogens. HIV-1 BG505 env served as negative control antigen and was not used for system-wide analyses.

**Fig 1:**
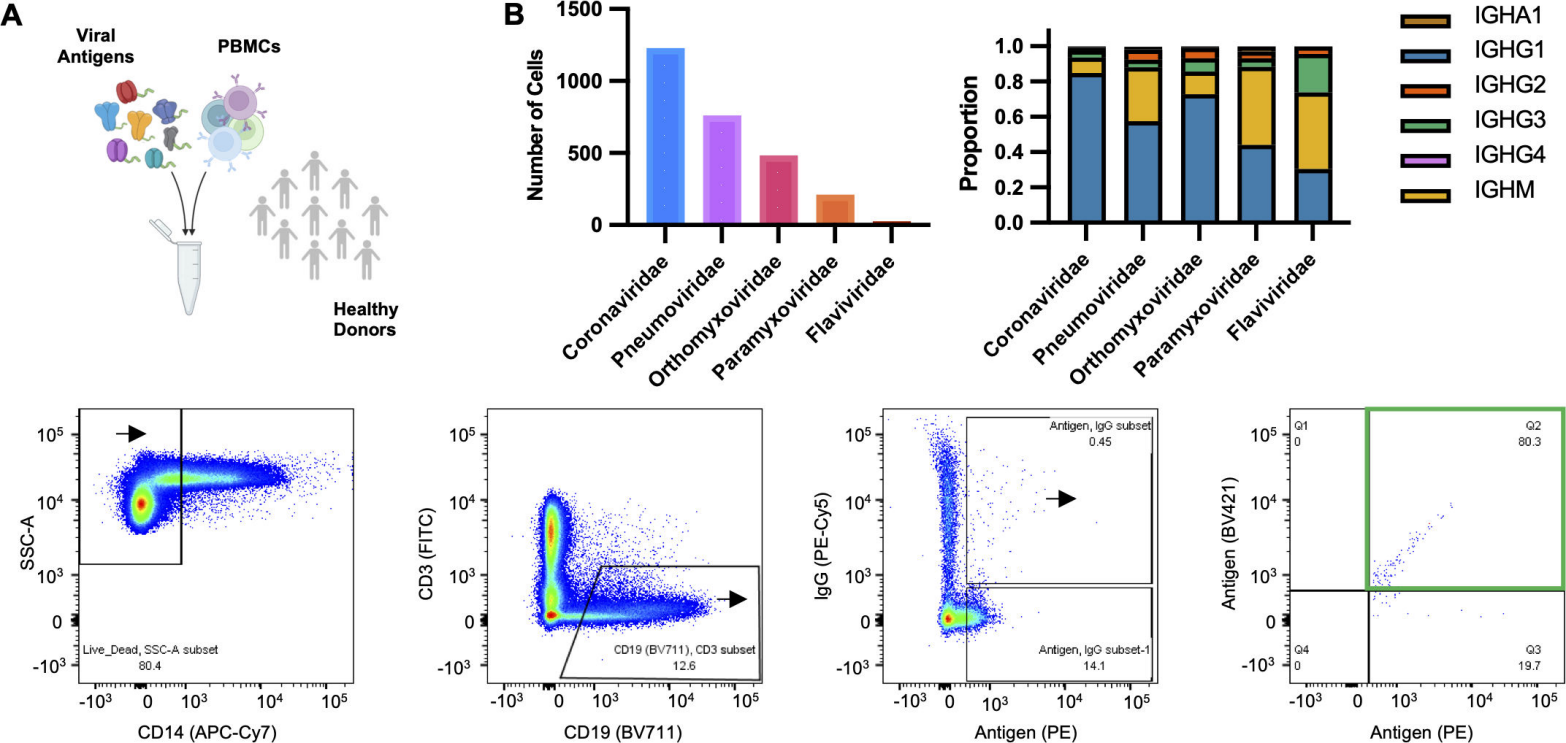
Identification of antigen-specific B cells from healthy donors. (**A**) Schematic of sample processing and flow cytometric staining to identify antigen specific B cells. Fluorescence-activated cell sorting of lymphocytes, B cells, and antigen-specific IgG cells. Cells were revealed by anti-CD14 allophycocyaninin cyanine dye (APC-Cy7), anti-CD19 brilliant violet 711 (BV711), anti-CD3 fluorescein isothiocyanate (FITC), and anti-IgG phycoerythrin cyanine dye (PE-Cy5). LIBRA-seq antigens were biotinylated and conjugated to streptavidin-phycoerythrin (Strep-PE) and streptavidin brilliant violet 421 (BV421). (**B**) Number of B cells identified for each antigen specificity category after computational filtering. Proportion of immunoglobulin isotypes identified within each antigen specificity category.

We sorted CD19^+^, IgG^+^, antigen^+^, B cells from 10 healthy donor PBMC samples using fluorescently tagged LIBRA-seq antigens as probes (Fig. 1A). Presumably, sorted cells are derived from the memory B-cell pool generated in response to infection by or vaccination with the antigens in the screening library. As memory B cells are the primary source of isotype-switched B-cell clones, and IgG is the predominant isotype in blood, we focused analysis on IgG^+^ cells, with expectations of recovering antigen-specific B cells for most of the antigens in the LIBRA-seq screening library. Resulting paired heavy and light chain sequences, along with LIBRA-seq scores which provide a relative measure of antigen binding, were analyzed as described in the methods. Antigen specificity was assigned based on the LIBRA-seq predictions, where a cell that displayed a LIBRA-seq score of one or greater for antigen X is considered to bind antigen X. After filtering the data for sequences that displayed promiscuous binding patterns, that is, binding to HIV-1 BG505 or binding to multiple antigens belonging to more than one viral family, we obtained a total of 2,686 BCR sequences with a LIBRA-seq score of at least one for a minimum of one antigen. Of those sequences, 2,101 were IgG restricted, dominated by the IgG1 subclass. Coronaviridae represented the largest group of antigen-specific B cells, followed by Pneumoviridae, Orthomyxoviridae, Paramyxoviridae, and Flaviviridae (Fig. 1B). The population of IgA and IgM recovered cells can be attributed to events that fall near the IgG^+^/PE-Cy5^+^ demarcation gate during flow cytometric sorting.

To provide a measure of the robustness of the LIBRA-seq antigen specificity predictions, we selected 69 paired heavy and light chain sequences from our filtered data set to express as monoclonal antibodies. B cells were prioritized based on binding profile and LIBRA-seq score. In addition, we selected 30 B cells from the subset of sequences that did not meet our filtering criteria (i.e., B cells with promiscuous binding patterns and/or B cells that did not meet additional filtering criteria as outlined in the methods), for a total of 99 antibodies for recombinant expression and validation. Of the 69 antibodies ordered from the filtered data sets, 59 of them bound to at least one of the antigens it was predicted to bind by LIBRA-seq, translating to an 85.5% true-positive rate. From the subset of sequences that did not meet filtering criteria, binding could be validated for 10 out of 30 antibodies. These sequences were therefore moved back to the filtered data set, indicating a 33% false-negative rate, reflective of the totality of the filtering system (Fig. S1).

### Antigen-specific patterns in V gene usage, IGHV:IGL(K)V gene pairings, CDRH3 length, and somatic hypermutation

**Fig 2:**
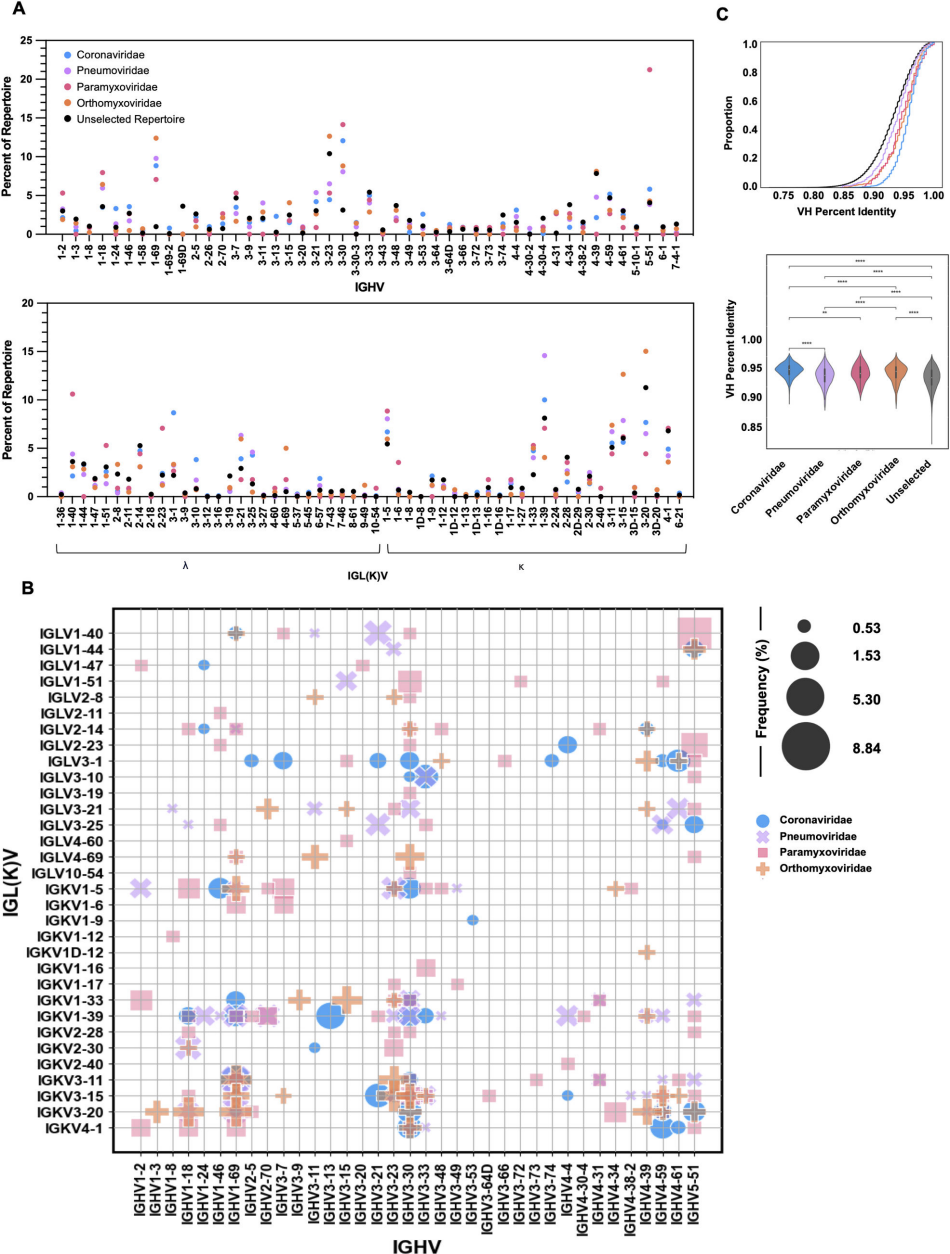
Diversity of viral antigen-specific B cells. (**A**) IGHV and IGL(K)V gene usage in B cells specific to Coronaviridae (blue), Pneumoviridae (purple), Paramyxoviridae (pink), and Orthomyxoviridae (orange) antigens. IGHV gene usage from previously published, unselected repertoires shown in black. Gene usage is represented as frequency of gene use within each antigen specificity category. Circles are superimposed. Zeros are not plotted. (**B**) The frequency of different IGHV:IGL(K)V gene pairs for B cells specific to Coronaviridae (blue circle), Pneumoviridae (purple cross), Paramyxoviridae (pink square), and Orthomyxoviridae (orange plus) antigens. The size of each data point represents the frequency of the corresponding IGHV:IGK(L)V pair within its antigen specificity category. Frequencies above 0.5% are represented. (**C**) Frequency of heavy chain variable (VH) somatic hypermutation represented as 1-VH identity calculated at the nucleotide level. Violin plot width is proportional to the fraction of B cells with the indicated proportion of VH somatic hypermutations. Two-sided Mann-Whitney-Wilcoxon test with Bonferroni correction used to calculate significance. Coronaviridae vs Pneumoviridae: *P* = 5.626e^−34^, Pneumoviridae vs Paramyxoviridae: *P* = 3.990e^−02^, Paramyxoviridae vs Orthomyxoviridae: *P* = 1.000 Coronaviridae vs Paramyxoviridae: *P* = 1.903e^−03^, Pneumoviridae vs Orthomyxoviridae: *P* = 2.173e^−06^, Coronaviridae vs Orthomyxoviridae: *P* = 2.751e^−07^. Orthomyxoviridae vs Unselected: *P* = 1.969e^−24^, Paramyxoviridae vs Unselected: *P* = 5.978e^−06^, Pneumoviridae vs Unselected *P* = 2.3853e^−05^, Coronaviridae vs Unselected: *P* = 1.522e^−157^.

We next explored the immunoglobulin heavy and light chain sequence features of the IgG-restricted sequences, excluding Flaviviridae reactive B cells due to insufficient numbers. As these are presumed to be healthy North American samples, B-cell reactivity to dengue antigens was not expected. Analysis revealed usage of 47 heavy chain, IGHV, and 56 light chain, IGL(K)V, variable genes, across the data set with several IGHV:IGL(K)V gene patterns occurring at a higher frequency within certain antigenic categories. For example, IGHV5-51:IGLV1-40 and IGHV5-51:IGLV2-23 were frequently observed within Paramyxoviridae and did not overlap with other specificity categories (Fig. 2A and B). In accordance with germline complexity of heavy chain variable segment families and published findings on VH bias of productive peripheral B cells (32, 33), all of the antigenic specificity categories exhibited a preference toward the VH3 family, while VH6 and VH7 were exceedingly rare (Fig. S2A). Notably, 21.2% of Paramyxoviridae-specific cells, across four donors, belonged to the VH5 family, an overrepresentation in comparison to the other categories. A consistent bias across all categories toward IGHJ4 was also observed, as demonstrated in previous reports of increased IGHJ4 gene usage as compared to other IGHJ genes (38) (Fig. S2B). IGHV1-69 has been implicated in several auto-immune and anti-pathogen repertoires (39–45) and indeed was observed among all specificity categories here. In comparing to previously published IgG repertoires, unselected by any antigen specificity (37), several genes were found at a higher frequency (defined as threefold greater) in one or more viral families, most notably IGHV1-69, IGHV3-30, and IGHV5-51 (Fig. 2A). Of note, previous pathogenic perturbation of the unselected repertoires is unknown. A similar distribution of heavy chain complementarity determining region 3 (CDRH3) lengths among the specificity categories was observed with Coronaviridae, Pneumoviridae, Orthomyxoviridae, and Paramyxoviridae having a majority of 16, 16, 18, and 12 amino acid long CDRH3 regions, respectively (Table 2; Fig. S3A). Finally, we evaluated the proportion of heavy chain variable (VH) somatic hypermutation, calculated as 1-VH identity at the nucleotide level, and found that the Pneumoviridae category displayed the highest frequency of VH somatic hypermutation among viral families, with an average VH identity of 0.93 and a range of 0.84–1.00 (Fig. 2C; Table 2). Expectedly, VH somatic hypermutation correlated with VL somatic hypermutation for all antigen specificity categories, as well as the unselected repertoire; Coronaviridae (*P* = 7.2e^−63^, Spearman 0.47), Pneumoviridae (*P* = 7.9e^−48^, Spearman 0.57), Paramyxoviridae (*P* = 2.1e^−21^, Spearman 0.57), Orthomyxoviridae (*P* = 8.7e^−47^, Spearman 0.62), Unselected (*P* < 0.0001, Spearman 0.67) (Fig. S3C).

**TABLE 2:** Sequence metrics of IgG restricted data sets^*a*^

Parameter	IgG restricted				IgG restricted by reactivity pattern	
	Coronaviridae	Pneumoviridae	Paramyxoviridae	Orthomyxoviridae	Mono-reactive	Cross-reactive
Total cells	1,091	492	107	398	1648	440
Mean CDRH3 length	17	17	16	18	17	17
Minimum CDRH3 length	7	7	8	8	7	8
Maximum CDRH3 length	37	29	27	30	37	30
Mode CDRH3 length	16	16	12	18	16	18
Mean VH percent identity	0.95	0.93	0.94	0.94	0.95	0.93
Minimum VH percent identity	0.85	0.84	0.87	0.84	0.84	0.84
Maximum VH percent identity	1.00	1.00	1.00	0.99	1.00	0.99
Mode VH percent identity	0.95	0.94	0.94	0.96	0.95	0.92
Number of donors represented	10	9	5	9		

^*a*^ Total cell counts, mean/minimum/maximum/mode heavy chain complementary determining region 3 (CDRH3) lengths, and mean/minimum/maximum/mode variable heavy (VH) somatic hypermutation (SHM) values for IgG restricted and reactivity pattern restricted data sets.

### Evaluation of the relationship between CDR3 sequence identity and antigen specificity of B cells

The antigen specificity of a B cell is determined by the B-cell receptor sequence, with (close to) identical sequences, that is, B cells of a shared clonotype, associated with similar specificities. Yet, it is not clear what level of sequence identity is needed for a pair of B cells in different donors to be considered public clones of each other, and therefore can be assumed to exhibit the same antigen specificity. To that end, we set out to interrogate the relationship between CDR3 identity in the heavy and light chains and the resulting antigen specificity in the B cells from the LIBRA-seq data sets, using previously reported definitions of public clonality (10, 11, 14, 18). CDRH3 sequence similarity among public clones defined in the literature ranged from 60% to 80% identity; therefore, we focused our analysis on pairs above a 70% CDRH3 identity threshold, as determined by calculating the Levenshtein distance where gaps in CDRH3 of different lengths are penalized the same as amino acid mismatches. Furthermore, analysis of D-gene segments was not considered due to the intrinsic nature of junctional processing that hinders accurate D-gene annotation.

**Fig 3:**
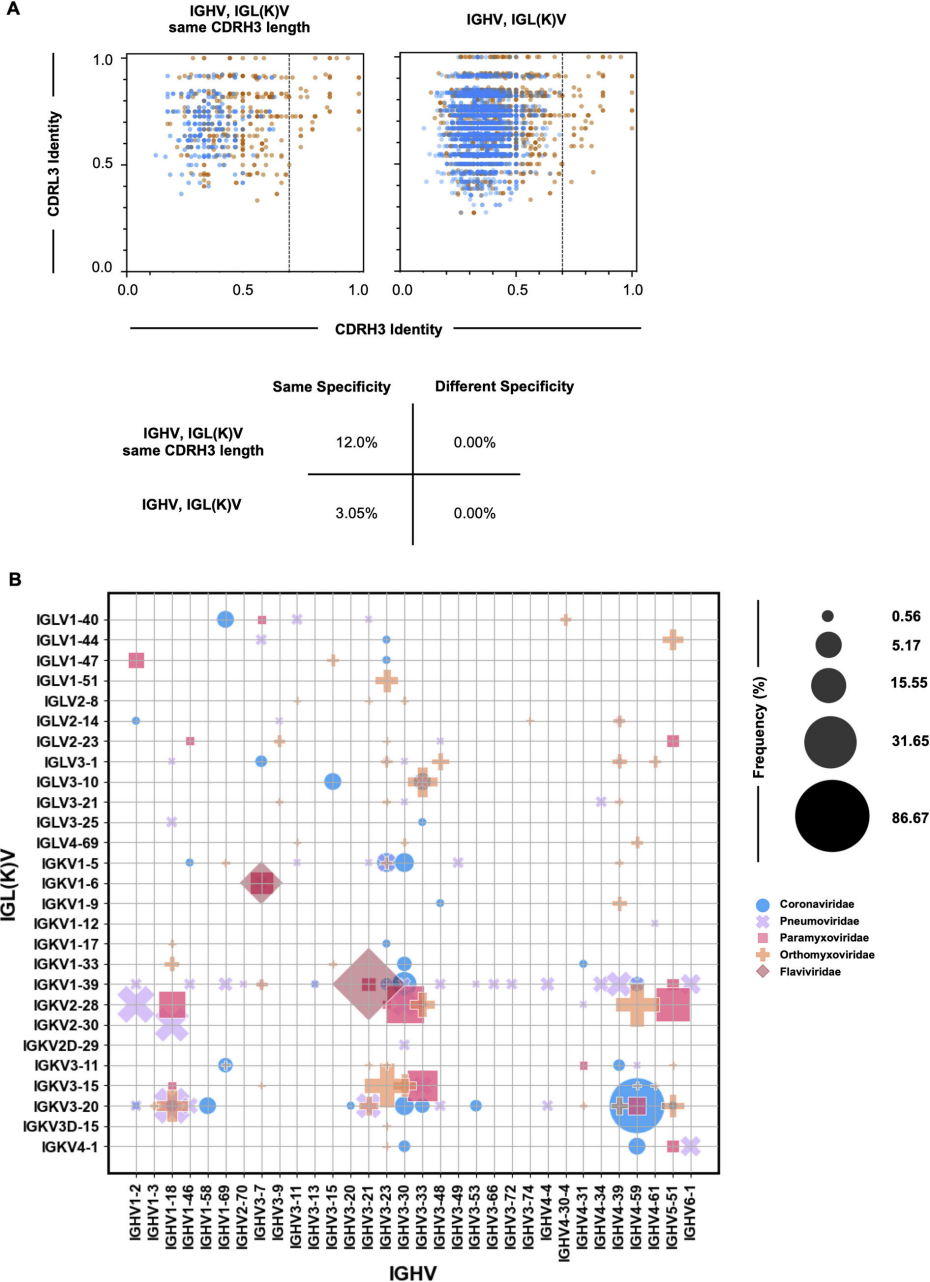
Germline gene usage and CDR3 identity thresholds among viral public clonotypes. (A) CDRH3 and CDRL3 identity in pairs of B cells encoded by identical IGHV and IGL(K)V genes with same length CDRH3 region (left) or identical IGHV and IGL(K)V genes without consideration of CDRH3 length (right). Pairs of B cells with the same antigen specificity are shown in orange; pairs of B cells with different antigen specificities are shown in blue. Identity calculated at the amino acid level. Matrix represents the percentage of events occurring with greater than or equal to 70% CDRH3 identity for both same and different antigen specificities with the listed genetic conditions. (B) The frequency of different IGHV:IGL(K)V gene pairs for predicted public clonotypes specific to Coronaviridae (blue circle), Pneumoviridae (purple cross), Paramyxoviridae (pink square), Orthomyxoviridae (orange plus), and Flaviviridae (red diamond) antigens. The size of each data point represents the frequency of the corresponding IGHV:IGK(L)V pair within its antigen specificity category. Frequencies above 0.5% are represented.

When comparing pairs of B cells originating from different donors but leveraging the same combination of V-genes, with or without consideration of J-genes (Fig. S4), in both the heavy and light chains, B-cell pairs displaying >70% CDRH3 identities were only identifiable when the B cells had the same antigen specificity (Fig. 3A), suggesting pairs of B cells under these specific indications representing different antigen specificities can share BCR sequence similarity only up to a specific sequence identity threshold, with BCRs greater than that threshold virtually guaranteed to have the same antigen specificity. Shared specificity does not appear to be dependent on matching CDRH3 length, whereby exclusion of CDRH3 length consideration increased the number of pairs with shared specificity, without breaching the 70% identity inflection point in pairs representing different specificities (Fig. 3A). When analysis was restricted to pairs of B cells where only the heavy, but not the light, V-genes were required to be the same, the inflection point in CDRH3 identity by which a pair of B cells can be assumed to share specificity exceeded 70% (Fig. S4). Interestingly, this observation in the CDRH3 inflection point remains true when identity was calculated at the nucleotide level, as opposed to amino acid level (Fig. S4), indicating under both circumstances, predicting shared specificity, without the potential for erroneous public antibody identification, requires greater than 70% CDRH3 identity. Sequence identity in the CDRL3 region appears indispensable under each of the defined similarity criteria. Together, these results suggest that for B cells originating from different donors but leveraging the same combination of, at a minimum, heavy and light chain V-genes and >70% CDRH3 identity, irrespective of matching CDRH3 length, could be a strong indicator of similarity in antigen specificity.

### Annotation of publicly available BCR data and identification of public viral antigen-specific B cells

To interrogate the existence of public antibodies within our data set, we built a reference library of paired BCR sequences from published sources (37, 46). The reference library contained 1,060,594 human sequences across five donor bins. Based on the gene usage considerations and CDR3 identity patterns identified in Fig. 3, that accurately predict shared specificity among pairs of B cells in different donors, sequences from the IgG restricted data set were screened for public-ness based on the following criteria: greater than 70% amino acid identity in the CDRH3 region and matching variable gene use in both heavy and light chains, irrespective of CDRH3 length. From the LIBRA-seq data sets, 391 sequences matched with 2,217 sequences in the reference library (Table S2), meeting the similarity criteria described above; these sequence matches were used for all subsequent public antibody analysis.

Despite the isolation of low cell numbers for Flaviviridae reactive cells, 13 public clonotypes were identified through our similarity criteria, and were therefore included in subsequent analysis. Of the Coronaviridae predicted public clonotypes identified, several of the heavy chain genes have previously been reported in SARS-CoV-2 public B cells: IGHV3-30 (12), IGHV3-53, IGHV1-58 (11), IGHV3-66 (30), IGHV1-69, IGHV4-59, and IGHV3-7 (10). Other previously identified public heavy chain genes in various anti-pathogen repertoires were also observed: IGVH 1–18 (21) and IGVH 1–69 (41) among influenza A hemagglutinin (HA) binding B cells, and IGVH 1-18, IGVH2-70, IGVH3-21, IGVH3-9, and IGVH 1–2 (17) within RSV repertoires. Analysis of IGHV:IGL(K)V gene pairing frequencies across the antigenic specificities revealed previously known gene usage patterns, such as IGHV 4-59: IGKV3-20 for SARS-CoV-2 (11) within Coronaviridae and IGHV 1-18: IGKV2-30 for RSV A/B (17) within Pneumoviridae, as well as enrichments for, to our knowledge, not yet reported, gene patterns and antigen specificities among public clonotypes, such as IGHV5-51: IGKV2-28, IGHV3-53: IGKV3-20, and IGHV1-18: IGKV2-28 within Paramyxoviridae, and IGHV 3-7:IGKV 1-6 within Flaviviridae (Fig. 3B). Collectively, these results demonstrate that public clonotypes of different antigen specificities can be readily identified and leverage distinct patterns in IGHV:IGL(K)V gene pairings.

### Mono-reactive and cross-reactive B cells display distinct patterns in V gene usage and somatic hypermutation

**Fig 4:**
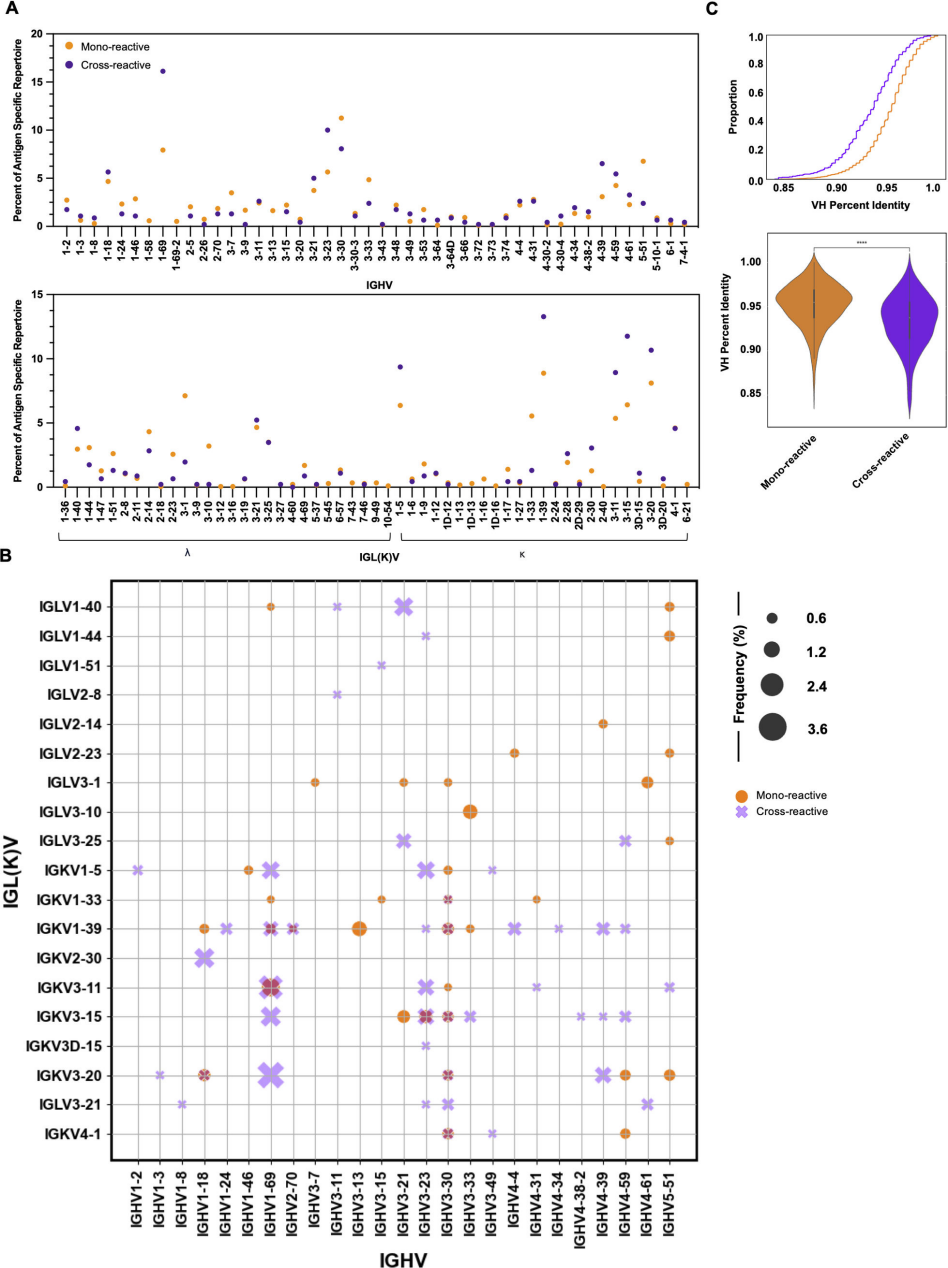
Molecular characteristics of mono-reactive and cross-reactive B cells. (A) IGHV and IGL(K)V gene usage in mono-reactive (orange) and cross-reactive (purple) B cells. Gene usage is represented as frequency of gene use within each reactivity category. Circles are superimposed. Zeros are not plotted. (B) The frequency of different IGHV:IGL(K)V gene pairs for B cells of mono- and cross-reactivity. The size of each data point represents the frequency of the corresponding IGHV:IGK(L)V pair within its reactivity category. Frequencies above 0.5% are represented. (C) Frequency of heavy chain variable (VH) somatic hypermutation represented as 1-VH identity calculated at the nucleotide level. Violin plot width is proportional to the fraction of antibodies with the indicated proportion of VH somatic hypermutation. Kruskal-Wallis test with Bonferroni correction used to calculate significance. *P* = 1.808^-36^.

Lastly, we investigated the relationship between the BCR sequence metrics observed above and B-cell breadth. Here, a mono-reactive B cell was categorized as displaying a minimum LIBRA-seq score of one for a single antigen, while a cross-reactive B cell was defined as displaying a minimum LIBRA-seq score of one for at least two antigens within the screening library. From the IgG-restricted dataset, 1,648 mono-reactive and 440 cross-reactive sequences were identified (Table 2). Cross-reactivity patterns were most often observed among antigens belonging to more closely related viruses (i.e., RSV A and RSV B, H1 NC99 and H1 MI15); however, more broadly cross-reactive B cells, such as those displaying binding to RSV and hMPV, diverse HA (H1, H3, H9), as well as pandemic (SARS-Cov-2) and endemic (HKU1, OC43) coronaviruses, were isolated (Fig. S1). While most IGHV and IGL(K)V genes were leveraged by both cell types, IGHV1-69 (16.1%) and IGHV3-30 (11.2%) were the most frequently observed IGHV genes for cross-reactive and mono-reactive cells, respectively, with IGKV1-39 (13.2% cross-, 8.8% mono-) observed most frequently for both categories (Fig. 4A). Analysis of IGHV:IGL(K)V revealed several gene pairs specific to either cell type, such as IGHV1-69:IGKV3-20 among the mono-reactive subset and IGHV3-33:IGLV3-10 among the cross-reactive subset (Fig. 4B). Interestingly, our analysis revealed cross-reactive B cells displayed higher degrees of VH (*P* = 1.808e^−36^) and VL (*P* = 3.353e^−18^) somatic hypermutation than mono-reactive B cells (Fig. 4C; Fig. S5A). As observed when analyzing somatic hypermutation with respect to antigen specificity in the IgG-restricted data set, VH somatic hypermutation moderately correlated with VL somatic hypermutation for both mono-reactive (*P* = 1.4e^−152^, Spearman 0.57) and cross-reactive cells (*P* = 1.2e^−36^, Spearman 0.54) (Fig. S5B). Analysis of VH percent identity across the most represented IGHV genes observed in both cell types revealed higher degrees of somatic hypermutation in several IGHV genes among cross reactive cells despite being used less frequently than mono-reactive cells (Fig. S5C).

## DISCUSSION

Leveraging LIBRA-seq, a high-throughput antibody discovery technology capable of screening a biological sample simultaneously against a large number of targets, we performed a systematic analysis of antigen-specific B-cell sequences against 20 viral antigens in healthy subjects. To facilitate systems-level analyses of the antigen specificity of antibody repertoires, we applied a number of stringent filtering criteria, including both from computational analysis and experimental feedback, with the primary goal of reducing the likelihood of false positive signals in the LIBRA-seq results. The inclusion of multiple pathogenic targets allowed us to explore patterns in several B-cell receptor sequence metrics in the context of viral antigen reactivity, revealing differences in germline gene usage, CDRH3 length, and degree of somatic hypermutation. Importantly, shared IGHV:IGL(K)V gene usage, with or without consideration of IGHJ:IGL(K)J usage, in combination with a 70% amino acid identity threshold in the CDRH3 region in pairs of B cells across donors appears to be a sufficient criterion for establishing similarity in antigen specificity of the B cells, as well as identifying public antibody clones. Through sequence analysis of the LIBRA-seq data sets, along with published antibody sequence data, we identified public antibody clonotypes against multiple targets, revealing convergent signatures in several antiviral pathogen repertoires. Together, our study represents the first of its kind to survey viral antigen-specific B cells at this scale, across multiple individuals, representing a crucial step forward in the generation of a comprehensive human antibody—viral antigen atlas.

Antigen specificity, indirectly afforded by BCR sequence, is the hallmark feature of antibodies. Identification of convergent BCR sequence motifs lends support to the indication that germline gene segments may have inherent properties that enable recognition of certain viral proteins, such as the interactions between the aromatic residues within IGHV1−58 + IGHJ3 and SARS-CoV-2 RBD F486 (29) and the hydrophobic loop of IGHV1-69 and the hydrophobic pocket of influenza HA stem (47). Yet still, the same IGHV:IGL(K)V combination can also be observed in multiple antigenic categories, suggesting that in some cases V-genes may exhibit a more promiscuous ability to adapt to recognition of diverse antigen targets. In these instances where a pair of B cells were encoded by the same IGHV:IGL(K)V combination, but recognized different antigenic targets, CDRH3 amino acid identity was not observed to exceed 70%.

In the absence of antigen specificity information, identifying public clonotypes may be used as a proxy for antigen specificity. However, there are a number of different definitions of what constitutes a public clonotype that vary in CDR3 sequence identity cutoff and heavy and light chain germline gene usage, fostering potential inaccuracies in public antibody identification. By contrast, our study directly interrogates the antibody sequence patterns associated with antigen specificity and provides experimentally grounded criteria in support of a 70% CDRH3 amino acid identity threshold irrespective of CDRH3 length, in pairs of B cells encoded by, at a minimum, identical IGHV:IGL(K)V genes, as a means of defining public clonality and therefore predicting B-cell antigen specificity in different individuals. Importantly, our results indicate that differential considerations in germline gene usage between pairs of B cells can be leveraged as an indicator of similarity in antigen specificity; however, the specific CDRH3 identity inflection point that governs whether a pair of B cells share specificity exhibits plasticity: as indicated by analysis of IGHV-gene similarity only or similarity in IGHV/IGL(K)V and IGHV/IGL(K)V/IGHJ/IGL(K)J gene usage when considering CDR3 identity at the nucleotide level, such definitions require greater than 70% CDRH3 identity thresholds to negate the potential for erroneous predictions in shared specificity between B-cell pairs. Of course, while it is possible to frequently observe B cells with CDR3 identities lower than the proposed thresholds that nevertheless exhibit the same antigen specificity, our analyses indicate that B cells with CDR3 identities greater than this threshold but with different antigen specificities may be exceedingly rare.

A holistic view of the molecular features underlying antibody reactivity has yet to be described; however, with continued technological advancements in B-cell receptor sequencing and the rise of *in silico* antibody engineering (48–50), antigen specificity prediction from antibody sequence alone is expected over time. The data presented here serve to enhance the space of antigen-annotated antibody sequence information, which is a current and major bottleneck for *in silico* modeling and prediction, as discovery efforts to date have been limited to individual pathogen targets, resulting in information for a handful of select antigens. We provide evidence that shared antigen specificity in pairs of B cells from different donors occurs under various considerations of germline gene use and CDR3 identity; however, careful consideration of CDRH3 sequence identity is requisite to avoid inaccurate antigen specificity assignment between pairs of B cells encoded by similar germline genes. This is of particular importance when leveraging public clonality as a means to annotate B-cell repertoire data lacking antigen specificity and functional characterization. Our analysis offers novel insights into canonical genetic features of population-level responses and may be used toward the rapid detection of antibodies of a shared reactivity profile from human samples. As evidenced by the COVID-19 pandemic and the critical need for the timely development of therapeutic countermeasures, the enhanced discovery of potential biologics in the wake of existing and emerging viral threats underscores the importance of understanding how antibody sequence influences antigen specificity.

## MATERIALS AND METHODS

### Healthy donor peripheral blood mononuclear cell samples

Peripheral blood mononuclear cell (PBMC) donor samples were procured from StemCell Technologies catalog number 70025. Donor PBMC samples were collected between June 2021 and May 2022. An equal distribution of men and women ages 23 through 59 was used in this study. Samples arrived frozen and were stored at −135°C until use. Donors are screened for HIV-1, HIV-2, hepatitis B, and hepatitis C within 90 days of collection.

### Cell lines

Ramos B-cell lines were engineered from a clone of Ramos Burkitt’s lymphoma that do not display endogenous antibodies, and they ectopically express specific surface IgM B-cell receptor sequences. The B-cell line used here expresses B-cell receptor sequences for HIV-specific antibody VRC01. The cells are cultured at 37°C with 5% CO_2_ saturation in complete RPMI, made up of RPMI supplemented with 15% fetal bovine serum, 1% L-Glutamine, and 1% Penicillin/Streptomycin.

### Antigen expression and purification

For the different LIBRA-seq experiments, a total of 21 proteins were expressed as recombinant soluble antigens.

Influenza, parainfluenza, HIV-1, and coronavirus antigens were expressed in Expi293F cells by transient transfection in FreeStyle F17 expression media (Thermo Fisher) supplemented to a final concentration of 0.1% Pluronic Acid F-68 and 20% 4 mM L-glutamine using Expifectamine transfection reagent (Thermo Fisher Scientific) cultured for 4–7 days at 8% CO_2_ saturation and 37°C with shaking. After transfection, cultures were centrifuged at 6,000 rpm for 20 min. The supernatant was filtered with Nalgene Rapid Flow Disposable Filter Units with PES membrane (0.45 or 0.22 µm), and then run slowly over an appropriate affinity column at 4C.

Previously described hMPV F A1(NL/1/00) and B2(TN99-419) antigens were expressed in FreeStyle 293-F cells by transient transfection in FreeStyle 293 expression media (Thermo Fisher). Cells were co-transfected at a 4:1 ratio of plasmids encoding human metapneumovirus F and furin, respectively, using polyethylenimine (PEI). Three hours post-transfection, medium was supplemented to a final concentration of 0.1% (vol/vol) Pluronic Acid F-68. After culturing for 6 days at 37°C and 8% CO_2_ saturation, the filtered supernatant was concentrated and the buffer was exchanged to PBS using tangential flow filtration. Samples were then run over a gravity-flow affinity column at room temperature. Previously described RSV F (DS-Cav1) A2 and B9320 antigens were expressed similarly but did not include the Pluronic F-68 supplementation step.

Single chain HIV-1 gp140 SOSIP variant strain BG505 (51) was purified over agarose-bound Galanthus nivalis lectin (Vector Laboratories cat no. AL-1243-5). The column was washed with PBS, and protein was eluted with 30 mL of 1 M methyl-a-D-mannopyranoside. The protein elution was buffer exchanged 3× into PBS and concentrated using 30 kDa Amicon Ultra centrifugal filter units. Concentrated protein was run on a Superose 6 Increase 10/300 Gl on the AKTA FPLC system. Peaks corresponding to trimeric species were identified based on elution volume and SDS-PAGE of elution fractions. Fractions containing pure HIV-1 BG505 Env were pooled.

Recombinant hemagglutinin (HA) proteins all contained the HA ectodomain with a point mutation at the sialic acid-binding site (Y98F), a T4 fibritin foldon trimerization domain, and a hexahistidine tag. HAs were purified by metal affinity chromatography. Tris (50 mM; pH 8.0) and 350 mM NaCl were added to the clarified supernatant using concentrated solutions of 1 M Tris (pH 8.0) and 5 M NaCl, respectively. For each liter of supernatant, 4 mL of Ni Sepharose Excel resin (GE) bed was washed with PBS using a gravity column and then added to the buffered supernatant, followed by overnight rocking at 4°C. The resin was collected 16–24 hours later using a gravity column, then washed once with 50 mM Tris (pH 8.0), 500 mM NaCl, 5 mM imidazole prior to elution of His-tagged protein using 50 mM Tris (pH 8.0), 500 mM NaCl, 300 mM imidazole. Eluates were concentrated and applied to a HiLoad 16/600 Superdex 200 pg column or a Superdex 200 Increase 10/300 Gl column pre-equilibrated with PBS for preparative size exclusion chromatography. Peaks corresponding to trimeric species were identified based on elution volume and SDS-PAGE of elution fractions. Fractions containing pure HA were pooled.

Parainfluenza virus type 3 prefusion stabilized F ectodomain (PDB: 6MJZ) was purified by nickel affinity chromatography using an equilibrated, 1 mL pre-packed Ni Sepharose High-Performance (HP) affinity resin (HisTrap HP) column (GE Healthcare, IL, USA). The column was washed with 15 mL of binding buffer (20 mM sodium phosphate, 0.5 M NaCl, 0.3 M imidazole, pH 7.4) and purified protein was eluted from the column with 15 mL of binding buffer supplemented with 0.5 M imidazole. Protein was concentrated, buffer exchanged into PBS, and run on a Superose 6 Increase 10/300 Gl on the AKTA FPLC system. Peaks corresponding to trimeric species were identified based on elution volume and SDS-PAGE of elution fractions. Fractions containing pure PIV-3 F were pooled.

SARS-CoV-2 S Hexapro Wuhan strain, HCoV-OC43 S, and HCoV-HKU1-S-2P were purified over equilibrated, 1 mL pre-packed StrepTrap XT column (Cytiva Life Sciences). The column was washed with 15 mL of binding buffer (100 mM Tris-HCl, 150 mM NaCl, 1 mM EDTA, pH 8.0), and purified protein was eluted from the column with 10 mL of binding buffer supplemented with 2.5 mM desthiobiotin. Protein was concentrated, buffer exchanged into PBS, and run on a Superose 6 Increase 10/300 Gl on the AKTA FPLC system. Peaks corresponding to trimeric species were identified based on elution volume and SDS-PAGE of elution fractions. Fractions containing pure spikes were pooled.

Stabilized ectodomains of hMPV F subtypes A1 and B2 (43), as well as RSV strains A2 (52, 53) and B9320 F (DS-Cav1) (54), were purified over Strep-Tactin Sepharose resin (IBA Lifesciences) in a gravity column. The resin was washed with four column volumes of PBS, and the purified protein was eluted from the column with four column volumes of 100 mM Tris (pH 8.0), 150 mM NaCl, 1 mM EDTA, and 2.5 mM desthiobiotin. Eluate was concentrated and run on a Superose 6 increase 10/300 Gl column pre-equilibrated with PBS (except for RSV A2 F, which was pre-equilibrated with 2 mM Tris [pH 8.0], 200 mM NaCl, and 0.02% NaN_3_) for preparative size exclusion chromatography. Peaks corresponding to trimeric species were identified based on elution volume and SDS-PAGE of elution fractions. Fractions containing pure fusion protein were pooled.

DENV-1, DENV-2, DENV-3, and DENV-4 E glycoproteins were recombinantly expressed and purified as previously described (55).

All proteins were quantified using UV/vis spectroscopy. Antigenicity of proteins was characterized by ELISA with known monoclonal antibodies specific for that antigen. Proteins were frozen and stored at −80°C until use.

### Biotinylation of antigens

Protein antigens were biotinylated using EZ-link Sulfo-NHS-Biotin No-Weigh kit (Thermo Fisher) according to the manufacturer’s instructions. A 50:1 biotin-to-protein molar ratio was used for all reactions.

### Oligonucleotide barcodes for LIBRA-seq

We used oligos that possess a 15 bp antigen barcode, a sequence capable of annealing to the template switch oligo that is part of the 10× bead-delivered oligos and contain truncated TruSeq small RNA read 1 sequences in the following structure: 5′-CCTTGGCACCCGAGAATTCCANNNNNNNNNNNNNNNCCCATATAAGA*A*A-3′, where Ns represent the antigen barcode. Oligos were ordered from Sigma-Aldrich and IDT with a 5′ amino modification and HPLC purified. The following antigen barcodes were used: CCGTCCTGATAGATG (HKU1), GTGTGTTGTCCTATG (OC43), TCACAGTTCCTTGGA (SARS-CoV-2), ACAATTTGTCTGCGA (RSV A), CAGGTCCCTTATTTC (RSV B), CAGCCCACTGCAATA (hMPV A), AACCTTCCGTCTAAG (hMPV B), CAGATGATCCACCAT (PIV-3), TACGCCTATAACTTG (H1 NC/99), TGGTAACGACAGTCC (H1 MI/15), AATCACGGTCCTTGT (H3 Perth), TCATTTCCTCCGATT (H3 HK/68), TCCTTTCCTGATAGG (H5 VN/04), CAGTAAGTTCGGGAC (H7 AN/13), ATTCGCCTTACGCAA (H9 HK/09), ATCGTCGAGAGCTAG (H10 JD/13), TGTGTATTCCCTTGT (DENV 1), CTTCACTCTGTCAGG (DENV 2), CAGTAGATGGAGCAT (DENV 3), GGTAGCCCTAGAGTA (DENV 4), and TAACTCAGGGCCTAT (HIV-1)

### Conjugation of oligonucleotide barcodes to antigens

For each antigen, a unique DNA barcode was directly conjugated to the antigen using a SoluLINK Protein-Oligonucleotide Conjugation kit (TriLink, S-9011) according to the manufacturer’s protocol. Briefly, the oligo and protein were desalted, and then the amino-oligo was modified with the 4FB crosslinker, and the biotinylated antigen protein was modified with S-HyNic. Then, the 4FB-oligo and the HyNic-antigen were mixed. This causes a stable bond to form between the protein and the oligonucleotide. The concentration of the antigen-oligo conjugates was determined by a BCA assay, and the HyNic molar substitution ratio of the antigen-oligo conjugates was analyzed using the NanoDrop according to the Solulink protocol guidelines. AKTA FPLC was used to remove excess oligonucleotide from the protein-oligo conjugates, which were also checked using SDS-PAGE with a silver stain.

### Flow cytometry enrichment of antigen-specific B cells

For a given sample, cells were mixed ~4% (of total viable PBMCs) with VRC01 Ramos cells, to serve as an internal negative control. Cell mixtures were stained and mixed with fluorescently labeled DNA-barcoded antigens and other antibodies, and then sorted using fluorescence-activated cell sorting (FACS). First, cells were counted, and viability was assessed using Trypan Blue. Then, cells were washed with DPBS supplemented with 0.1% Bovine serum albumin (BSA) through centrifugation at 300 g for 5 minutes. Cells were resuspended in DPBS-BSA and stained with a variety of cell markers. These markers included Ghost Red 780, CD14-APCCy7, CD3-FITC, CD19-BV711, and IgG-PECy5. In addition, antigen-oligo conjugates were added to the stain. After a 30-min incubation in the dark on ice, the cells were washed again three times with DPBS-BSA at 300 × *g* for 5 min. Then, the cells were incubated for 15 min in the dark on ice with streptavidin-PE and streptavadin-BV421 to label cells with bound antigens. Cells were then resuspended in DPBS-BSA and sorted on the cell sorter. Antigen-positive cells were bulk sorted and then delivered to the Vanderbilt VANTAGE sequencing core at an appropriate target concentration for 10x Genomics library preparation and subsequent sequencing. FACS data were analyzed using FlowJo.

### 10x Genomics single-cell processing and next-generation sequencing

Single-cell suspensions were loaded onto the Chromium Controller microfluidics device (10x Genomics) and processed using the B cell Single Cell V(D)J solution according to the manufacturer’s suggestions for a target capture of 10,000 B cells per 1/8 10× cassette for B cells. Slight modifications were made to intercept, amplify, and purify the antigen barcode libraries, as previously described (24).

### Sequence processing and bioinformatics analysis

We followed our established pipeline, which takes paired-end FASTQ files of oligonucleotide libraries as input, to process and annotate reads for cell barcodes, unique molecular identifiers (UMIs), and antigen barcodes, resulting in a cell barcode-antigen barcode UMI count matrix (24). B-cell receptor contigs were processed using CellRanger 3.1.0 (10x Genomics) and GRCh38 Human V(D)J 7.0.0 as a reference, while the antigen barcode libraries were also processed using CellRanger (10x Genomics). The cell barcodes that overlapped between the two libraries formed the basis of the subsequent analysis. Cell barcodes that had only non-functional heavy chain sequences as well as cells with multiple functional heavy chain sequences and/or multiple functional light chain sequences, were eliminated, reasoning that these may be multiplets. We also aligned the B-cell receptor contigs (filtered_contigs.fasta file output by CellRanger, 10x Genomics) to IMGT reference genes using HighV-Quest (56). The output of HighV-Quest was parsed using ChangeO (57) and combined with an antigen barcode UMI count matrix. Finally, we determined the LIBRA-seq score for each antigen in the library for every cell, as previously described (24). Briefly, starting with the UMI count matrix, we first filter for noise and set low-level UMI counts (1–3) to 0. We then add a pseudo count of 1 and take the centered log ratios of each antigen UMI count for each cell before taking the Z score of the transformed data. Lastly, we set all scores of 0 to the minimum score for that antigen.

### LIBRA-seq filtering pipeline

Prior to filtering, 44,275 sequences were recovered from the raw Cell Ranger VDJ output. To adjust for noise due to ambient antigen barcode capture and non-specific binding interactions, a negative binomial mixture model was developed to calculate the probability of a given UMI count being considered signal or noise. This approach leveraged the VRC01-BG505 negative control system to fit a mixed model for the distribution of UMI counts for each antigen within each sample. For each antigen, the distribution of UMI counts for the antigen bound to VRC01 was assumed to be technical noise. VRC01 cells were selected using a 95% identity threshold to the VRC01 CDRH3 nucleotide sequence using Levenshtein distance. A mixed binomial distribution then was fit to the distribution of UMIs representing binding to the donor (non-VRC01) cells for that antigen. After fitting, probability mass functions (PMF) were then generated from the optimized parameters, and Baye’s theorem was used to calculate the probability of each UMI count being in the “signal” component of the mixed distribution. This is an adaptation of a mixed Gaussian model, using the negative binomial distributions to cluster the UMI counts into each of the two distributions. Finally, the probability of being signal was used for filtering, with a 90% probability threshold included in addition to the LIBRA-seq score (LSS) threshold of 1. VRC01 cells were not recovered from donor 3 (8365-3), and this sample therefore did not utilize this mixed modeling filtering approach.

After generating probabilities of UMI counts being signaled in the raw counts using the mixed model described above, the following filtering steps were implemented to further filter the cells using a custom Python script. VRC01 cells were removed, along with cells that bound to the BG505 negative control antigen (LSS ≥ 1). To account for technical artifacts resulting from LSS calculated for antigens with very low counts across all cells, UMI counts that were less than 10 but yielded LSS ≥1 were set to the minimum LSS for that antigen. For defining binding families and type specificities, binding was defined as having a LSS ≥1. Cells that bound to more than one viral family were considered polyreactive and removed, along with cells with LSS <1 for all antigens in the experiment. Furthermore, within a given donor, clonal relatives that exhibited inconsistent LIBRA-seq scores compared to other cells in the same clone were removed. Only IgG isotype cells were retained for the systematic repertoire (non-public) analyses. Following *in vitro* validation by ELISA, filtering thresholds were modified to inform the systems and clonotype analysis. For specific antibodies tested by ELISA, LSS for these cells was updated to reflect the assay results. A further UMI threshold for Dengue antigens (DENV E 3 and DENV E 4) was also implemented due to the high false-positive LSS rate observed, with UMI counts less than 400 for DENV E 3 and less than 120 for DENV E 4 considered non-binding.

### V(D)J sequence analysis

After pre-processing and filtering, the V(D)J sequences and annotations were further analyzed using custom scripts written in Python 3.10, utilizing NumPy 1.23 and Pandas 1.4.3 for data wrangling, Matplotlib 3.6.2 and Seaborn 0.12.2 for visualization, and Scipy 1.8.1

### Public antibody analysis

The reference library combined paired heavy chain-light chain human BCR sequences from published sources (37, 46). We used custom Python scripts utilizing NumPy and Pandas to determine public clones between our data and the reference library. Public clones were identified on the basis of greater than 70% amino-acid sequence identity in CDRH3 and matching heavy and light variable (V) gene usage. Levenshtein distance was used for comparison in CDR3 identities and when comparing CDRH3 of different lengths, gaps are penalized the same as mismatches.

### High-throughput recombinant monoclonal antibody microexpression

Microscale transfection was performed with Expi293F cells in FreeStyle F17 expression media (Thermo Fisher) supplemented to a final concentration of 0.1% Pluronic Acid F-68 and 20% 4 mM L-glutamine using Expifectamine transfection reagent (Thermo Fisher Scientific). Briefly, synthesized antibody-encoding DNA from Twist BioSciences (0.8 µg per transfection) was added to OptiMem directly, incubated with ExpiFectamine transfection reagent, and added to 800 µL of Expi293 cell cultures in deep 96-well blocks. The plates were incubated on an orbital shaker at 900 rpm with an orbital diameter of 3 mm at 37°C in 8% CO_2_. Culture supernatants were collected at day five post-transfection. Blocks were centrifuged at max speed and supernatant was carefully removed and immediately used for ELISA.

### Enzyme-linked immunosorbent assay

Soluble protein was plated at 2 μg/mL overnight at 4°C. The next day, plates were washed three times with PBS supplemented with 0.05% Tween20 (PBS-T) and coated with 1% BSA in PBS-T. Plates were incubated for 1 hour at room temperature and then washed three times with PBS-T. Supernatant from antibody microscale transfection was then plated and incubated at room temperature for 1 h, and then washed three times in PBS-T. The secondary antibody, goat anti-human IgG conjugated to peroxidase, was added at 1:10,000 dilution in 1% BSA in PBS-T to the plates, which were incubated for 1 hour at room temperature. Plates were washed three times with PBS-T and then developed by adding TMB substrate to each well. The plates were incubated at room temperature for 5 min, and then 1 N sulfuric acid was added to stop the reaction. Plates were read at 450 nm. ELISAs were repeated two or more times.

## Data Availability

Raw sequencing data have been deposited to Sequence Read Archive and processed data to Gene Expression Omnibus (PRJNA1049361).

## SUPPLEMENTAL MATERIAL

Supplemental material mbio.01560-24-s0001.pdf Supplemental figures and tables.

ASM does not own the copyrights to Supplemental Material that may be linked to, or accessed through, an article. The authors have granted ASM a non-exclusive, world-wide license to publish the Supplemental Material files. Please contact the corresponding author directly for reuse.
