# Supplementary material for: Antibody sequence determinants of viral antigen specificity
Source: mBio. 2024 Sep 12;15(10):e01560-24. doi: 10.1128/mbio.01560-24 (PMC11481873; doi:10.1128/mbio.01560-24)
Supplement: Supplemental material — Supplemental figures and tables. [file mbio.01560-24-s0001.pdf]

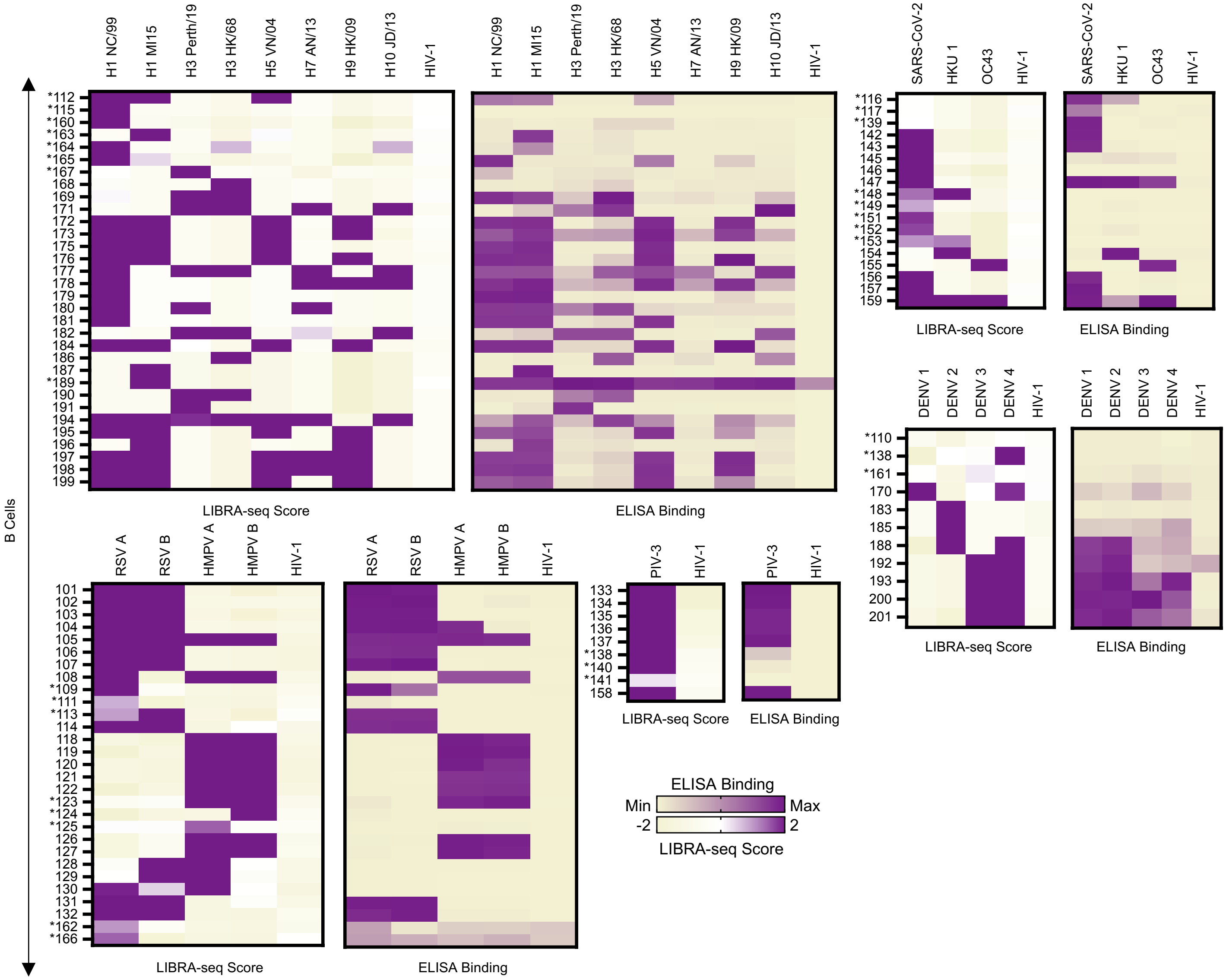
B Cells
H1 NC/99
H1 MI15
H3 Perth/19
H3 HK/68
H5 VN/04
H7 AN/13
H9 HK/09
H10 JD/13
HIV-1
*112 *115 *160 *163 *164 *165 *167 168 169 171 172 173 175 176 177 178 179 180 181 182 184 186 187 *189 190 191 194 195 196 197 198 199
LIBRA-seq Score
ELISA Binding
SARS-CoV-2
HKU 1
OC43
HIV-1
*116 *117 *139 142 143 145 146 147 *148 *149 *151 *152 *153 154 155 156 157 159
LIBRA-seq Score
ELISA Binding
DENV 1
DENV 2
DENV 3
DENV 4
HIV-1
*110 *138 *161 170 183 185 188 192 193 200 201
LIBRA-seq Score
ELISA Binding
RSV A
RSV B
HMPV A
HMPV B
HIV-1
101 102 103 104 105 106 107 108 *109 *111 *113 114 118 119 120 121 122 *123 *124 *125 126 127 128 129 130 131 132 *162 *166
LIBRA-seq Score
ELISA Binding
PIV-3
HIV-1
133 134 135 136 137 *138 *140 *141 158
LIBRA-seq Score
ELISA Binding
ELISA Binding
Min
Max
-2
2
LIBRA-seq Score

Figure S1.

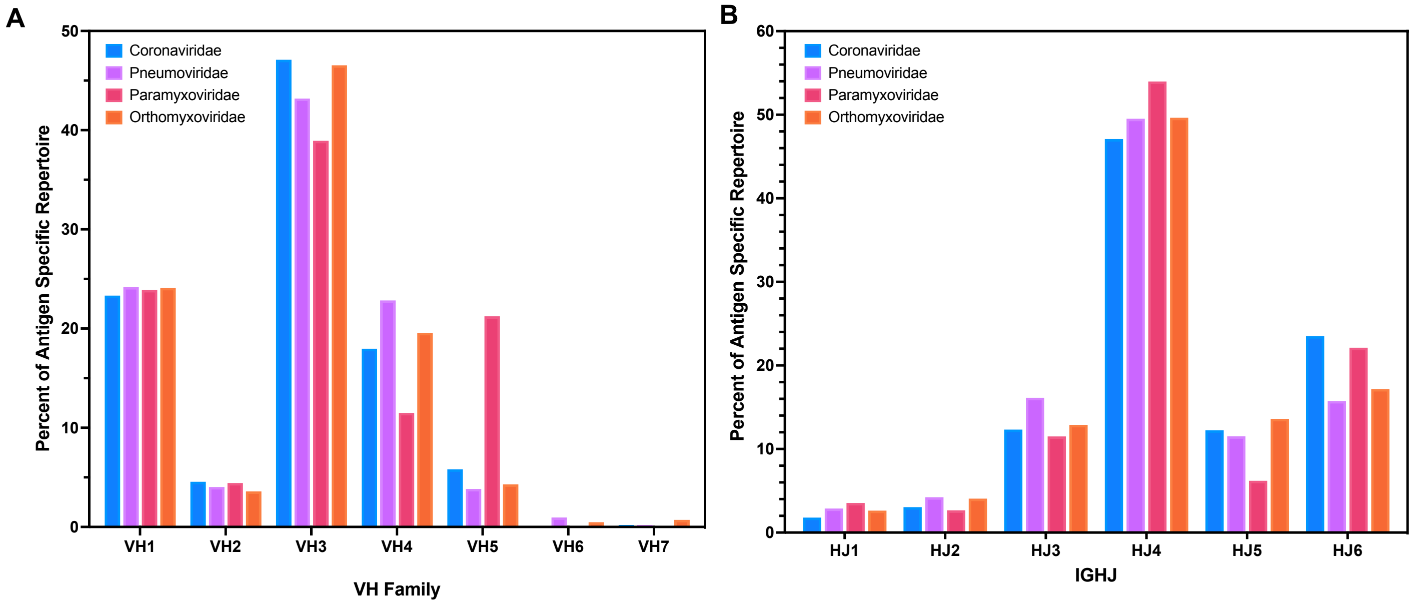
A
Coronaviridae
Pneumoviridae
Paramyxoviridae
Orthomyxoviridae
Percent of Antigen Specific Repertoire
0
10
20
30
40
50
VH1
VH2
VH3
VH4
VH5
VH6
VH7
VH Family
B
Coronaviridae
Pneumoviridae
Paramyxoviridae
Orthomyxoviridae
Percent of Antigen Specific Repertoire
0
10
20
30
40
50
60
HJ1
HJ2
HJ3
HJ4
HJ5
HJ6
IGHJ

Figure S2.

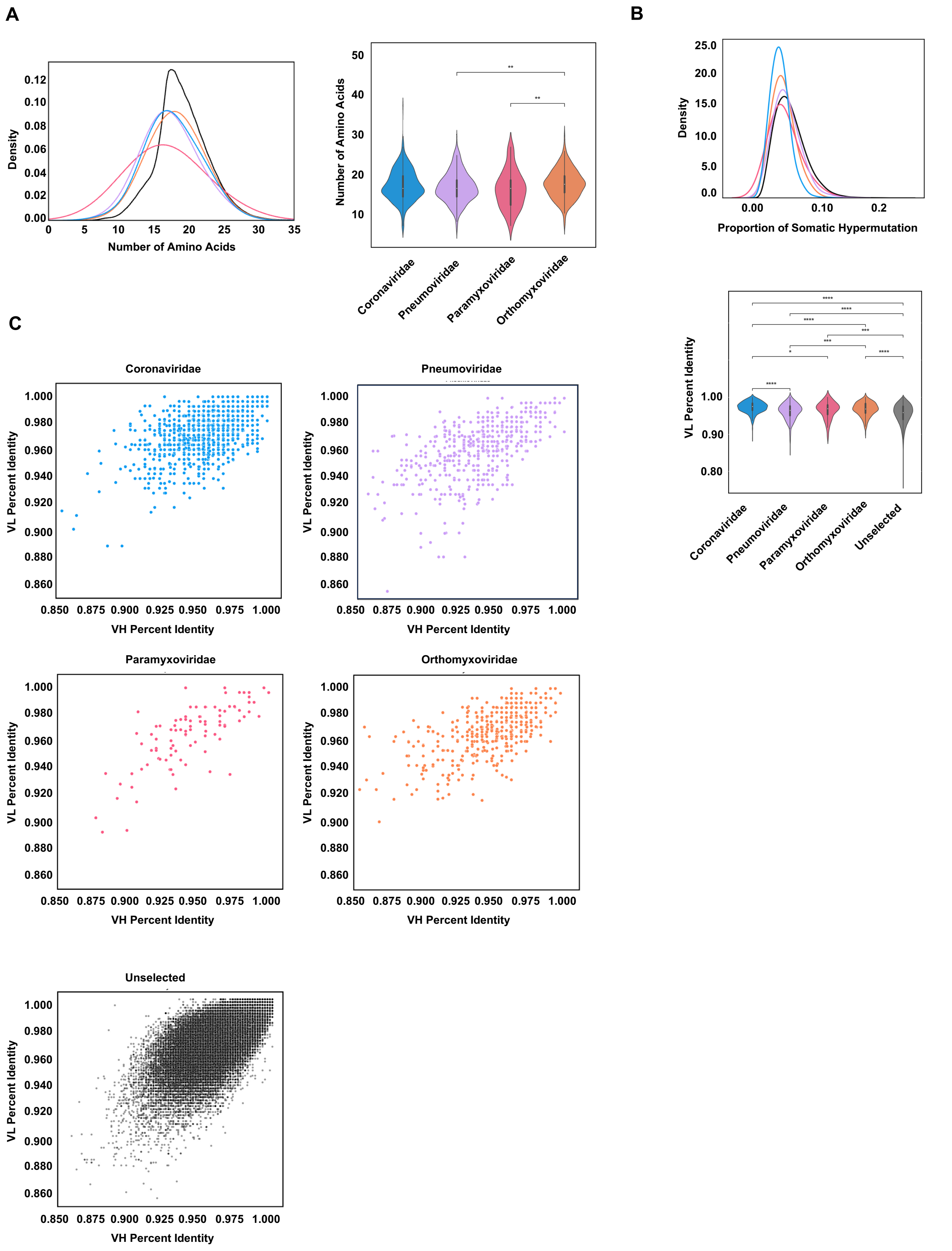
A
Density
Number of Amino Acids
Number of Amino Acids
Coronaviridae
Pneumoviridae
Paramyxoviridae
Orthomyxoviridae
B
Density
Proportion of Somatic Hypermutation
VL Percent Identity
Coronaviridae
Pneumoviridae
Paramyxoviridae
Orthomyxoviridae
Unselected
C
Coronaviridae
Pneumoviridae
Paramyxoviridae
Orthomyxoviridae
Unselected
VL Percent Identity
VH Percent Identity

Figure S3.

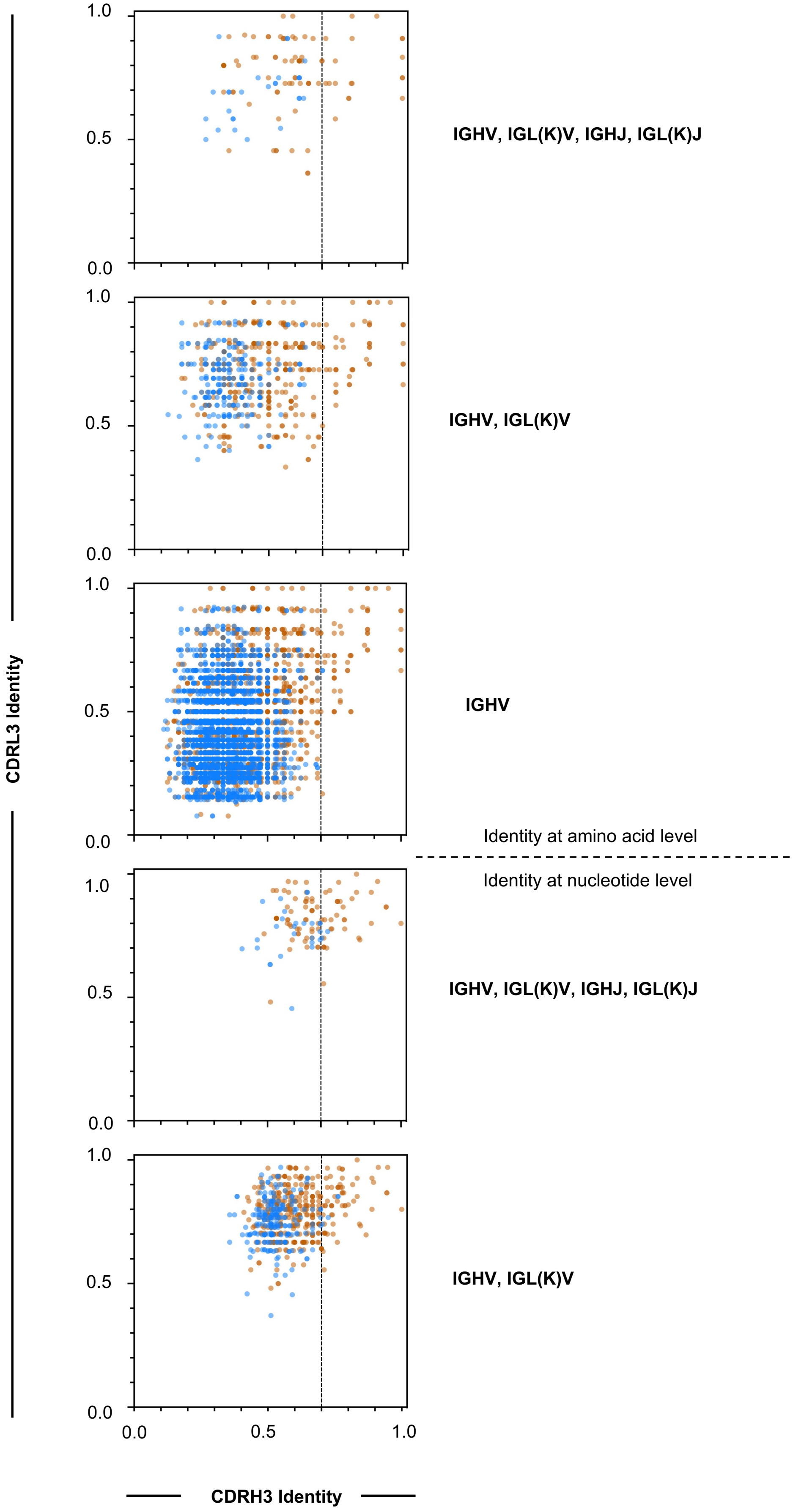
CDRL3 Identity
CDRH3 Identity
IGHV, IGL(K)V, IGHJ, IGL(K)J
IGHV, IGL(K)V
IGHV
Identity at amino acid level
Identity at nucleotide level
IGHV, IGL(K)V, IGHJ, IGL(K)J
IGHV, IGL(K)V
0.0
0.5
1.0

Figure S4.

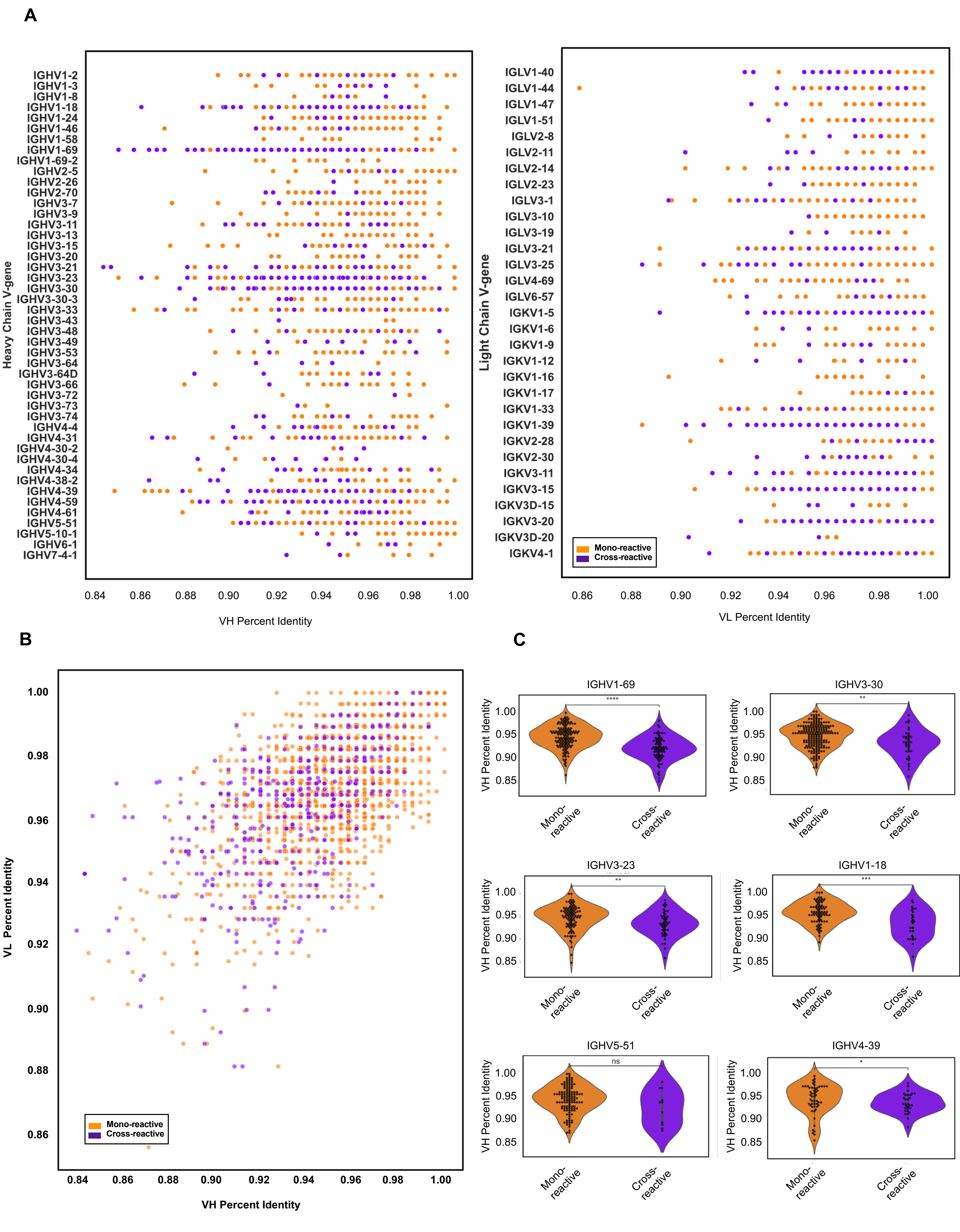
A
Heavy Chain V-gene
IGHV1-2
IGHV1-3
IGHV1-8
IGHV1-18
IGHV1-24
IGHV1-46
IGHV1-58
IGHV1-69
IGHV1-69-2
IGHV2-5
IGHV2-26
IGHV2-70
IGHV3-7
IGHV3-9
IGHV3-11
IGHV3-13
IGHV3-15
IGHV3-20
IGHV3-21
IGHV3-23
IGHV3-30
IGHV3-30-3
IGHV3-33
IGHV3-43
IGHV3-48
IGHV3-49
IGHV3-53
IGHV3-64
IGHV3-64D
IGHV3-66
IGHV3-72
IGHV3-73
IGHV3-74
IGHV4-4
IGHV4-31
IGHV4-30-2
IGHV4-30-4
IGHV4-34
IGHV4-38-2
IGHV4-39
IGHV4-59
IGHV4-61
IGHV5-51
IGHV5-10-1
IGHV6-1
IGHV7-4-1
0.84 0.86 0.88 0.90 0.92 0.94 0.96 0.98 1.00
VH Percent Identity
Light Chain V-gene
IGLV1-40
IGLV1-44
IGLV1-47
IGLV1-51
IGLV2-8
IGLV2-11
IGLV2-14
IGLV2-23
IGLV3-1
IGLV3-10
IGLV3-19
IGLV3-21
IGLV3-25
IGLV4-69
IGLV6-57
IGKV1-5
IGKV1-6
IGKV1-9
IGKV1-12
IGKV1-16
IGKV1-17
IGKV1-33
IGKV1-39
IGKV2-28
IGKV2-30
IGKV3-11
IGKV3-15
IGKV3D-15
IGKV3-20
IGKV3D-20
IGKV4-1
Mono-reactive
Cross-reactive
0.86 0.88 0.90 0.92 0.94 0.96 0.98 1.00
VL Percent Identity
B
VL Percent Identity
1.00 0.98 0.96 0.94 0.92 0.90 0.88 0.86
Mono-reactive
Cross-reactive
0.84 0.86 0.88 0.90 0.92 0.94 0.96 0.98 1.00
VH Percent Identity
C
IGHV1-69
****
IGHV3-30
**
IGHV3-23
**
IGHV1-18
***
IGHV5-51
ns
IGHV4-39
*
VH Percent Identity
1.00 0.95 0.90 0.85
Mono-reactive
Cross-reactive

Figure S5.

Dataset	Cell Processing Date	Age	Sex	Ethnicity
8365-1	9/1/21	55	M	Caucasian
8365-2	7/1/21	37	M	Caucasian
8365-3	9/2/21	50	F	Caucasian
8365-4	6/2/21	59	M	Caucasian
8365-5	6/8/21	46	M	Caucasian
8365-6	6/7/21	46	F	Caucasian
8365-8	6/28/21	28	M	Caucasian
8365-9	6/15/21	23	F	Caucasian
8365-13	5/2/22	23	F	African American
8365-19	7/27/21	25	F	Caucasian

Table S1: Donor peripheral mononuclear blood cell (PBMC) data.

	Coronaviridae	Pneumoviridae	Paramyxoviridae	Orthomyxoviridae	Flaviviridae
Reference Library	1557	227	118	302	13
LIBRA-seq datasets	244	63	17	65	2

Table S2: Identified public clonotypes.

SUPPLEMENTARY FIGURE/ TABLE TITLES AND LEGENDS

Figure S1: Validation of LIBRA-seq predicted antigen-specific B cells.
LIBRA-seq scores (left) and ELISA binding (right) of recombinantly expressed monoclonal antibodies, partitioned by viral family. Antibodies are numbered 100-201. Antibodies denoted with asterisk originate from the unfiltered dataset. Some antibodies with asterisk are listed twice if binding predictions span viral families. LIBRA-seq scores for each antigen are displayed as a heatmap with a LIBRA-seq score of −2 displayed as beige, 0 as white, and a LIBRA-seq score of 2 as purple; scores lower or higher than that range are shown as −2 and 2, respectively. ELISA binding, calculated as absorbance at 450 nm, is displayed as a heatmap with the minimum detected signal (0.03) displayed as beige and maximum detected signal (3.5) displayed in purple.

Figure S2: Variable (V) and joining (J) gene segment usage frequencies.
A: Observed frequencies of V gene families from the IgG restricted dataset, represented as frequency of segment use within each antigen specificity category.
B: Observed frequencies of J genes families from the IgG restricted dataset, represented as frequency of segment use within each antigen specificity category.

Figure S3: CDRH3 length and somatic hypermutation of viral antigen specific B cells.
A: CDRH3 length represented as the frequency within each antigen specificity category. Previously published, unselected repertories are shown in black. Violin plot width is

proportional to the fraction of B cells with the indicated CDRH3 length. Two sided Mann-Whitney-Wilcoxon test with Bonferroni correction used to calculate significance. Coronaviridae vs. Pneumoviridae: p= 1.038e^{-01}, Pneumoviridae vs. Paramyxoviridae: p= 8.767e^{-01}, Paramyxoviridae vs. Orthomyxoviridae: p= 4.052e^{-03}, Coronaviridae vs. Paramyxoviridae: p= 5.075e^{-02} , Pneumoviridae vs. Orthomyxoviridae: p= 1.292e^{-03}, Coronaviridae vs. Orthomyxoviridae: p= 3.050e^{0}.

B: Frequency of light chain variable (VL) somatic hypermutation represented as 1-VL identity calculated at the nucleotide level. Violin plot width is proportional to the fraction of B cells with the indicated proportion of VL somatic hypermutations. Two sided Mann-Whitney-Wilcoxon test with Bonferroni correction used to calculate significance. Coronaviridae vs. Pneumoviridae: p= 1.118e^{-23}, Pneumoviridae vs. Paramyxoviridae: p= 2.870e^{-01}, Paramyxoviridae vs. Orthomyxoviridae: p= 1.000, Coronaviridae vs. Paramyxoviridae: p= 1.636e^{-02}, Pneumoviridae vs. Orthomyxoviridae: p= 4.598e^{-04}, Coronaviridae vs. Orthomyxoviridae: p= 2.601e^{-06}, Orthomyxoviridae vs. Unselected: p= 1.219e^{-20}, Paramyxoviridae vs. Unselected: p= 1.828e^{-04}, Pneumoviridae vs. Unselected p= 2.386e^{-06}, Coronaviridae vs. Unselected: p= 7.076e^{-126}.

C: Correlation between the proportion of heavy chain variable gene somatic hypermutation (x axis) and light chain variable gene somatic hypermutation (y axis) of B cells for each antigen specificity category. Coronaviridae spearman correlation coefficient 0.47 p = 7.2^{-63}. Pneumoviridae spearman correlation coefficient 0.57 p= 7.9e^{-48}. Paramyxoviridae spearman correlation 0.74 p= 2.1e^{-21}. Orthomyxoviridae spearman correlation coefficient 0.62 p=8.7e^{-47}. Unselected spearman correlation coefficient 0.67 p= <0.0001.

Figure S4: Analysis of LIBRA-seq datasets using previously reported definitions of public clonality.

CDRH3 and CDRL3 identity in pairs of B cells encoded by shared germline genes with shared specificities or different specificities. (Top down) Pairs of B cells with identical IGHV, IGL(K)V, IGHJ, IGL(K)J; IGHV, IGL(K)V; IGHV genes. CDR3 identity calculated at the amino acid level. Pairs of B cells with identical IGHV, IGL(K)V, IGHJ, IGL(K)J; IGHV, IGL(K)V genes. CDR3 identity calculated at the nucleotide level. For all indications, pairs of B cells were encoded by the same number of amino acids in the CDRH3 region. Pairs of B cells with the same antigen specificities are shown in orange. Pairs of B cells with different antigen specificities are shown in blue.

Figure S5: Variable gene somatic hypermutation of mono-reactive and cross-reactive B cells.

A: Dot plot indicating VH percent identity for IGHV genes leveraged by mono-reactive and cross-reactive cells. Dot plot indicating VL percent identity for IGL(K)V genes leveraged by mono-reactive and cross-reactive cells.

B: Correlation between the VH percent identity (x axis) and VL percent identity (y axis). Mono-reactive spearman correlation coefficient 0.57, p= $1.4e^{-152}$. Cross-reactive spearman correlation coefficient 0.54, p=$1.2e^{-36}$.

C: VH percent identity for the most represented IGHV genes leveraged by both mono-reactive and cross-reactive cells. Kruskal- Wallis test with Bonferroni correction used to calculate significance. In order of most represented to least: IGHV1-69, IGHV3-30,

IGHV3-23, IGHV1-18, IGHV5-51, IGHV4-39. IGHV1-69 p = 1.584^{-10}, IGHV3-30 p = 1.388^{-03}, IGHV3-23 p = 4.028^{-03}, IGHV1-18 p = 3.367^{-04}, IGHV5-51 p = 1.319^{-01}, IGHV4-39 = 4.476^{-02}

Table S1: Donor peripheral mononuclear blood cell (PBMC) data.

PBMC samples were procured from StemCell Technologies. Donor cell processing date, age, sex, and ethnicity are listed for each donor.

Table S2: Identified public clonotypes.

Number of sequences identified as public from the reference library and the LIBRA-seq datasets are shown for each antigen specificity category.
